# Study of polymorphisms in *tir*, *eae *and *tccP2 *genes in enterohaemorrhagic and enteropathogenic *Escherichia coli *of serogroup O26

**DOI:** 10.1186/1471-2180-11-124

**Published:** 2011-05-30

**Authors:** Marjorie Bardiau, Sabrina Labrozzo, Jacques G Mainil

**Affiliations:** 1 Bacteriology, Department of Infectious and Parasitic Diseases, Veterinary Faculty, University of Liège, Liège B4000, Belgium

## Abstract

**Background:**

Enteropathogenic (EPEC) and enterohaemorrhagic (EHEC) *Escherichia coli *are responsible for food poisoning (enteritis and enterotoxaemia) in humans in developed countries. Cattle are considered to be an important reservoir of EHEC and EPEC strains for humans. Moreover, some of the strains, belonging to the O26, O111, O118 serogroups, for example, are also responsible for digestive disorders in calves. The Translocated intimin receptor (Tir), the intimin (Eae) and the Tir-cytoskeleton coupling protein (TccP) represent three virulence factors implicated in the intimate attachment of the bacteria to the eukaryotic cell. Major variants have already been described for these genes among the different serogroups but minor variations have not often been studied. In this study, we examined the polymorphisms of the *tir*, *eae *and *tccP2 *genes of O26 strains (EPEC and EHEC isolated from bovines and from humans) with the aim to determine whether these polymorphisms are host specific or not.

**Results:**

Of the 70 tested strains, 10 strains (14% of the strains) presented one or several polymorphisms in the *tir *and *eae *genes, which have never previously been described. Concerning *tccP2 *detection, 47 of the 70 strains (67% of the strains) were found to be positive for this gene. Most of the strains were found to possess *tccP2 *variants described in strains of serogroup O26. Nevertheless, three strains had *tccP2 *genes respectively described in strains of serogroup O111, O103 and O55. Moreover, none of the polymorphisms was statistically specific to the bovine or the human isolates. Nevertheless, the two major variants of *tccP2 *were statistically associated with the pathotype (EPEC or EHEC).

**Conclusions:**

In conclusion, *tir *and *eae *gene polymorphisms were found not to be numerous and not to be predominantly synonymous. Moreover, no difference was observed between human and bovine strains regarding the presence of polymorphisms. Finally, some *tccP2 *variants appeared to be pathotype specific. Further investigations need to be performed on a larger number of strains in order to confirm this specificity.

## Background

Enteropathogenic (EPEC) and enterohaemorrhagic (EHEC) *Escherichia coli *represent two important classes of enteric pathogens. EPEC strains belonging to different serogroup (e.g. 026, 055, 086, 0111, O128) are a major cause of infant diarrhoea in many countries and are also associated with diarrhoea in most domestic animal species [1,2]. These strains can be classified into two groups: typical-EPEC strains (t-EPEC), harbouring a specific plasmid named EPEC Adherence Factor (EAF plasmid), and atypical-EPEC strains (a-EPEC), which do not carry this specific EAF plasmid. EHEC strains have been responsible for individual cases, and small to large outbreaks in developed countries [3-8]. O157:H7 is the main serotype responsible for human illness in several countries. Nevertheless non-O157 serogroups can also be associated frequently with severe disease in humans and O26 serogroup represent the second more important serogroup in Europe [9-11]. Syndromes caused in humans are diverse: undifferentiated diarrhoea, haemorrhagic colitis (HC), haemolytic uremic syndrome (HUS) and thrombotic thrombocytopaenic purpura (TP) [12]. Transmission often occurs via consumption of foodstuffs contaminated by faeces from ruminants (mainly cattle), which can be asymptomatic healthy carriers [13,14]. Nevertheless, several serogroups of EHEC strains (e.g. O26, O111, O118) are also associated with diarrhoea in calves [15-18].

EPEC and EHEC share four stages in their pathogenicity: (1) colonisation of the intestine by specific adhesins, (2) translocation of a signal into the enterocyte by the type III secretion system (T3SS) of the bacteria and integration of the Translocated intimin receptor (Tir) into the host cell membrane, (3) intimate adhesion of bacteria to eukaryote cells by specific adhesins (intimins) that bind to Tir, and (4) actin polymerization after Tir phosphorylation. These four stages allow the bacteria to produce a specific lesion called an "attaching and effacing (A/E) lesion" [1]. Furthermore, as well as using the Tir phosphorylation pathway, some strains (EPEC 2 strains and the vast majority of non-O157 EHEC strains) are able to utilize the T3SS effector TccP2 (Tir-cytoskeleton coupling protein 2) to trigger actin polymerization, which leads to the formation of a pedestal characteristic of the A/E lesion [19].

Sequence variations in the Tir receptor-, intimin adhesin- and TccP2 effector-encoding genes (*tir*, *eae *and *tccp2*) have been described between EHEC and EPEC strains, and these can lead to major or minor polymorphisms (variants) of the encoded proteins [20-24]. Major variants of Tir and intimin are related, to some extent, to the serogroups of the EHEC and EPEC strains, whereas minor variants can exist within a serogroup for the same major variant, although these have not often been defined [25,26]. EHEC and EPEC strains belonging to the O26 serogroup classically produce the beta major variant of Tir and intimin, but their minor variants have not been studied [26,27]. Only two major variants of TccP have been described that are related to the pathotype of the strain [19]. EHEC and EPEC strains of O26 serogroup produce the TccP2 variant with six minor variants identified [23,24].

The purposes of this study were (1) to investigate the polymorphism of the *tir*, *eae *and *tccP2 *genes between O26 EPEC and EHEC strains isolated from bovines and from humans; and (2) to determine whether these polymorphisms are specific to bovine or human strains.

## Results

### Detection of *tir*, *eae *and *tccP2 *genes

All the tested strains of serogroup O26 were found to possess β type *eae *and *tir *genes. Moreover, of the 70 tested strains, 10 strains (14% of the strains) presented one or several polymorphisms in these two genes. None of the polymorphic strains possessed polymorphism in both *eae *and *tir *genes. Concerning *tccP2 *detection, 47 of the 70 strains (67% of the strains) were positive for this gene. Most of the strains possessed *tccP2 *variants described in strains of serogroup O26. Three strains had *tccP2 *genes respectively described in strains of serogroup O111, O103 and O55.

### Polymorphisms in the *eae *gene

For the *eae *gene, four polymorphisms were detected in nucleotide positions 255 (G > A), 1859 (C > T), 2415 (A > T) and 2772 (C > T) in *eae β *gene reference strain 14I3, (accession number FJ609815) and five unique *eae β *genotypes were defined (Table 1). The "classical" genotype (strain 14I3 sequence) was represented by 93% (65+/70) of the strains and the four other genotypes were represented by only one or two strains. Even though there was no statistical significance (p = 0.078), all the strains that presented polymorphism were bovine EPECs. One polymorphism was non-synonymous and gave one genotype different in the amino-acid (AA) sequence: valine was coded in place of alanine in AA position 620. This AA is situated in the D0 Ig-like domain.

**Table 1:** *eae β *gene polymorphism (aa: amino acid, A: alanine, V: valine)

	Number of strains			Polymorphism 1	Polymorphism 2	Polymorphism 3	Polymorphism 4
				(S) 255 G => A	(NS) 1859 C => T	(S) 2415 A => T	(S) 2772 C => T
	Human	Bovine			620 aa: A =>V		
	0	1	Genotype 1	+	-	-	-
	0	2	Genotype 2	+	+	-	-
	0	1	Genotype 3	-	-	+	-
	0	1	Genotype 4	-	-	-	+
	28	37	Genotype 5	-	-	-	-
						
**Total**	**28**	**42**					

### Polymorphisms in the *tir *gene

For the *tir *gene, five polymorphisms were detected in nucleotide positions 133 (T > G), 571 (insertion of GATACAAAG), 939 (G > A), 1080 (G > T) and 1302 (C > T) in *tir β *genes reference strain 95ZG1 (accession number AF070068) and four unique *tir β *genotypes were defined (Table 2). Interestingly, one polymorphism (position 939) was found to be present in all the strains. One genotype was represented by 93% (65+/70) of the strains, and the other three genotypes were represented by only one or two strains. Two polymorphisms were found to be non-synonymous and gave three different genotypes in the AA sequences: for the first polymorphism, serine was coded in place of alanine in AA position 45; for the second polymorphism, three AA (TKE) were inserted into AA position 191. These two polymorphisms were situated in the N-terminal part of the gene. Nevertheless, when we compared polymorphisms regarding the host and the pathotype (EPEC or EHEC), none was found to be specific to the bovine or the human isolates (p < 0.05) or to EPEC or EHEC pathotype.

**Table 2:** *tir β *gene polymorphism (aa: amino acid, A: alanine, S: serine, T: threonine, K: lysine, E: glutamic acid)

	Number of strains			Polymorphism 1	Polymorphism 2	Polymorphism 3	Polymorphism 4	Polymorphism 5
				(S) 1080 G => T	(S) 1302 C => T	(NS) 133 T => G	(NS) insertion1 571 GATACAAAG	(S) 939 G => A
	Human	Bovine				45 aa: S => A	191 aa: TKE	
	0	2	Genotype 1	+	-	-	-	+
	2	0	Genotype 2	-	+	-	+	+
	1	0	Genotype 3	-	-	+	-	+
	25	40	Genotype 4	-	-	-	-	+
							
**Total**	**28**	**42**						

### Polymorphisms in the *tccP2 *gene

For the *tccP2 *gene, seven genotypes (Table 3) were detected in the collection. All had been previously described [23,24]. The *tccP2 *variant described in reference strain 11368 (accession number AB253564) was found to be present in 34% (24+/70) of the strains. The *tccP2 *variant described in reference strain EC38/99 (accession number AB275131) was present in 17% (12+/70) of the strains. *tccP2 *variants described in reference strains 12009 and CB00225 (accession number AB253581 and AB275122 respectively) were both present in 6% (4+/70) of the strains. Three *tccP2 *variants described in reference strains ED411, ED71 and 5905 (accession number AB253567, AB253576 and AB356001 respectively) were represented by only one strain each. None of the variants was found to be specific to the bovine or the human isolates (p < 0.05). Nevertheless, the two major variants were statistically associated with the pathotype (p < 0.01): the *tccP2 *gene AB275131 was statistically associated with the EPEC strains in comparison with the EHEC strains and the *tccP2 *gene AB253564 was statistically associated with the EHEC strains in comparison with the EPEC strains.

**Table 3:** *tccP2 *gene polymorphism

Accession number of *tccP2 *variant (variant described in serogroup)	Positive isolates in				
	
	Total	EHEC	EPEC	Bovine	Human
**AB253564 (O26)**	**24+/70**	21+/44	3+/26	17+/42	7+/28
**AB275131 (O26)**	**12+/70**	2+/44	10+/26	9+/42	3+/28
**AB275122 (O26)**	**4+/70**	0+/44	4+/26	4+/42	0+/28
**AB253581 (O013)**	**4+/70**	4+/44	0+/26	0+/42	4+/28
**AB253567 (O26)**	**1+/70**	1+/44	0+/26	0+/42	1+/28
**AB253576 (O55)**	**1+/70**	1+/44	0+/26	1+/42	0+/28
**AB356001 (O111)**	**1+/70**	0+/44	1+/26	0+/42	1+/28

## Discussion

The Tir receptor (encoded by the *tir *gene) and the intimin adhesin (encoded by the *eae *gene) are both implicated in the adherence of the EPEC and EHEC strains to eukaryotic cells via the binding of the intimin to the Tir receptor (previously translocated to the host cell). The A/E lesion is then produced and is characterized by the loss of microvilli and intimate attachment of the bacteria to the host cell. Moreover, non-O157 strains can utilize TccP2, as well as Tir, to trigger actin polymerization during the production of the A/E lesion [19]. There are variations in the *eae*, *tir *and *tccP2 *gene sequence and many variants have been described [20-22]. Nevertheless small variations (polymorphisms) inside the same variants have not often been described. In 2007, Bono *et al*.[25] studied the polymorphism of *tir *and *eae *genes in O157 strains and associated two tir polymorphisms with the isolate source (bovine or human). With this in mind, we performed the present work to study the polymorphism of the *tir*, *eae *and *tccP2 *genes existing in O26 EPEC and EHEC strains isolated from bovines and from humans with a view to determinate whether these polymorphisms are specific to bovine or human strains in the O26 serogroup.

*tccP2 *variants were found to be present in 67.1% of the tested strains. This is not surprising regarding the results obtained by Ooka *et al*. and Ogura *et al*., who respectively found the *tccP2 *gene in 82.3% of O26 a-EPEC strains and in 71.4% of O26 EHEC strains [23,24]. It is possible that the negative isolates use only the Tir phosphorylation pathway or that they utilize another unknown pathway. Moreover, the distribution of *tccP2 *variants appears to be specific to the pathotype. In our study, *tccP2 *variant (accession number AB253564) originally described in the O26 EHEC 11368 reference strain was found to be statistically associated to EHEC strains in our study and *tccP2 *variant (accession number AB275131) originally described in O26 a-EPEC EC38/99 reference strain was found to be statistically associated to a-EPEC strains. However, further studies need to be performed in order to confirm this pathotype specificity. If this association appears to be confirmed, it could be used as a tool to study, among other things, O26 EPEC strains (isolated from patients or from calves) in order to determine if these strains are "real" O26 EPEC strains or O26 EHEC strains that have lost *stx *genes[28].

In comparison with O157 strains, O26 strains do not possess a large number of polymorphisms in the *tir *gene (only four different genotypes were revealed by our study and the major one was represented by 92.8% of the strains in comparison with ten different genotypes revealed by the study of Bono *et al*. with the major one represented by 68.6%). By contrast, *eae *polymorphisms are, in both studies, very limited. Bono *et al*. explained this difference in polymorphism frequency (between *eae *and *tir *genes) among O157 strains by the fact that both genes have evolved under a different kind of selective pressure. The difference in *tir *polymorphism frequency between O157 and O26 strains could also be explained by a different kind of selective pressure between both serogroups. Currently, we know that O157 EHEC strains and O26 EHEC and EPEC strains possess two different actin signalling pathways [19]. The O157 EHEC strains use only the TccP adaptor to induce actin polymerization and the O26 EHEC and EPEC strains can use two other pathways: the TccP2 adaptor and the phosphorylation of Y474 Tir residue. Therefore, it is not surprising that *tir *polymorphisms are more frequent in O157 EHEC strains than in O26 EHEC and EPEC strains.

Furthermore, the polymorphisms in *tir *and *eae *genes revealed by our study are mainly synonymous. For the *eae *gene, only one polymorphism was found to be non-synonymous (valine is coded in place of alanine in position 620) and this is situated in the D0 Ig-like domain. This polymorphism is not surprising and the consequences on the protein structure are probably nil for two reasons: firstly, in the *eae *ζ gene, valine is situated at this position and secondly, D0 is a divergent region that is not entirely conserved [29]. For the *tir *gene, two polymorphisms were found here to be non-synonymous and these are located near the amino terminus of Tir. This region is normally situated in the host cytosol after Tir translocation and is probably implicated in pedestral length, pedestral efficiency and translocation in the host cell [30].

Finally, concerning host specificity, in contrast to O157 strains [25], our study revealed that *tir *and *eae *polymorphisms are not associated with the host (human or bovine). In comparison to O157 strains, which seem to be host classifiable using nucleotide polymorphisms [31,32], we were unable to distinguish O26 strains. Several studies have suggested that O157 strains can be separated into two distinct lineages (lineages I and II), which appear to have distinct ecological characteristics, and which are associated with the host [33-36].

## Conclusions

In conclusion, *tir *and *eae *genes of O26 EHEC and EPEC strains are well conserved. Polymorphisms are not numerous or predominantly synonymous. Moreover, no difference was observed between human and bovine strains regarding the presence of polymorphisms. Finally, *tccP2 *variants appear to be pathotype specific. Further investigations need to be performed on a larger number of strains in order to confirm this specificity.

## Methods

### Bacterial strains

A total of 70 EHEC (n = 44) and EPEC (n = 26) strains of serogroup O26 isolated from bovine (n = 42) and humans (n = 28) and from diverse countries (USA, Ireland, Belgium, France, Japan and Brazil) were studied. Most of the strains had been described previously [37,38] and their pathotype (EPEC or EHEC) and serotype O26:H11 had been confirmed by PCR for *stx1, stx2, eae, wzx-wzy_O26 _*and *fliC_H11 _*genes [39-41].

### PCR reaction

A 2941 pb segment of the *eae *gene, a 1559 pb segment of the *tir *gene and a 753 pb segment of *tccP2 *gene were amplified by PCR, using respectively four pairs of primers, two pairs of primers and one pair of primers. All the primers used in this study and all the annealing temperatures are listed in Table 4. For PCR reactions, the following mixture was used: 1 U of *Taq *DNA polymerase (New England Biolabs, USA), 5 μl of 2 mM deoxynucleoside triphosphates, 5 μl of 10X ThermoPol Reaction Buffer (20 mM Tris-HCl (pH 8.8, 25°C), 10 mM KCl, 10 mM (NH_4_)_2_SO_4_, 2 mM MgSO_4_, 0.1% Triton X-100), 5 μl of each primer (10 μM), and 3 μl of a DNA template in a total volume of 50 μl.

**Table 4:** Primers used in this study (R = A+G, K = T+G, Y = C+T)

Primer name	Sequence (5' to 3')	Target gene	Annealing temp. (°C)	Amplicon size (bp)	Reference
**B52**	AGGCTTCGTCACAGTTG	*eaeA*	50	570	[39]
**B53**	CCATCGTCACCAGAGGA				
					
**B54**	AGAGCGATGTTACGGTTTG	*stx1*	50	388	[39]
**B55**	TTGCCCCCAGAGTGGATG				
					
**B56**	TGGGTTTTTCTTCGGTATC	*stx2*	50	807	[39]
**B57**	GACATTCTGGTTGACTCTCTT				
					
**wzx-wzyO26-F**	AAATTAGAAGCGCGTTCATC	*wzx_O26_*	56	596	[41]
**wzx-wzyO26-R**	CCCAGCAAGCCAATTATGACT				
					
**fliC-H11-F**	ACTGTTAACGTAGATAGC	*fliC_H11_*	56	224	[41]
**fliC-H11-R**	TCAATTTCTGCAGAATATAC				
					
**B139**	CRCCKCCAYTACCTTCACA	*tir β*	53	560	[27]
**B140**	GATTTTTCCCTCGCCACTA				
					
**tir(591-1617)-F**	TCCAAATAGTGGCGAGGGAA	*tir β*	54	1026	This study
**tir(591-1617)-R**	TTAAACGAAACGTGCGGGTC				
					
**B73**	TACTGAGATTAAGGCTGATAA	*eae β*	50	520	[27]
**B137**	TGTATGTCGCACTCTGATT				
					
**eae(37-1142)-F**	CGGCACAAGCATAAGCTAAA	*eae β*	51	1105	This study
**eae(37-1142)-R**	AGTTTACACCAACGGTCGCC				
					
**eae(1001-2046)-F**	TCCGCTTTAATGGCTATTTACC	*eae β*	50	1045	This study
**eae(1001-2046)-R**	TGCCTTCGCTGTTGTTTTAT				
					
**eae(2319-2972)-F**	GGCTCTGCAAAGAACTGGTT	*eae β*	50	653	This study
**eae(2319-2972)-R**	AGTCTCTATCAAACAAGGATACACG				
					
**tccP2-F**	ATGATAAATAGCATTAATTCTTT	*tccP2*	56	753	[24]
**tccP2-R**	TCACGAGCGCTTAGATGTATTAAT				

### DNA sequencing

The DNA fragments amplified were purified using the NucleoSpin Extract II kit (Macherey-Nagel, Germany) according to the manufacturer's instructions. Sequencing of the two DNA strands was performed by the dideoxynucleotide triphosphate chain termination method with a 3730 ABI capillary sequencer and a BigDye Terminator kit version 3.1 (Applied Biosystems, USA) at the GIGA (Groupe Interdisciplinaire de Génoprotéomique Appliquée, Belgium). Sequence analysis was performed using Vector NTI 10.1.1 (Invitrogen, USA). DNA sequencing was performed three times.

### Statistical analysis

A Fisher's exact test was performed to assess statistical differences.

## Authors' contributions

MB conceived of the study, carried out the sequence alignment and drafted the manuscript. SL carried out the PCR reactions. JGM participated in the design and coordination of the study and helped to draft the manuscript. All authors read and approved the final manuscript.
